# Clinical outcome and predictors of survival and pneumonitis after stereotactic ablative radiotherapy for stage I non-small cell lung cancer

**DOI:** 10.1186/1748-717X-7-152

**Published:** 2012-09-10

**Authors:** Joe Y Chang, Hui Liu, Peter Balter, Ritsuko Komaki, Zhongxing Liao, James Welsh, Reza J Mehran, Jack A Roth, Stephen G Swisher

**Affiliations:** 1 Departments of Radiation Oncology, The University of Texas MD Anderson Cancer Center, 1515 Holcombe Blvd., Houston, TX, USA; 2 Department of Radiation Physics, The University of Texas MD Anderson Cancer Center, Houston, TX, USA; 3 Department of Thoracic and Cardiovascular Surgery, The University of Texas MD Anderson Cancer Center, Houston, TX, USA

**Keywords:** Stereotactic body radiotherapy, Stereotactic ablative radiotherapy, Non-small cell lung cancer, F-fluorodeoxyglucose positron emission tomography, Toxicity, Predictive factors

## Abstract

**Background:**

Stereotactic ablative radiotherapy (SABR) can achieve excellent local control rates in early-stage non-small cell lung cancer (NSCLC) and has emerged as a standard treatment option for patients who cannot undergo surgery or those with isolated recurrences. However, factors that may predict toxicity or survival are largely unknown. We sought here to identify predictors of survival and pneumonitis after SABR for NSCLC in a relatively large single-institution series.

**Methods:**

Subjects were 130 patients with stage I NSCLC treated with four-dimensional computed tomography (4D CT) –planned, on-board volumetric image–guided SABR to 50 Gy in 4 fractions. Disease was staged by positron emission tomography/computed tomography (PET/CT) and scans were obtained again at the second follow-up after SABR.

**Results:**

At a median follow-up time of 26 months, the 2-year local control rate was 98.5%. The median overall survival (OS) time was 60 months, and OS rates were 93.0% at 1 year, 78.2% at 2 years, and 65.3% at 3 years. No patient experienced grade 4–5 toxicity; 15 had radiation pneumonitis (12 [9.3%] grade 2 and 3 [2.3%] grade 3). Performance status, standardized uptake value (SUV)_max_ on staging PET/CT, tumor histology, and disease operability were associated with OS on univariate analysis, but only staging SUV_max_ was independently predictive on multivariate analysis (*P* = 0.034). Dosimetric factors were associated with radiation pneumonitis on univariate analysis, but only mean ipsilateral lung dose ≥9.14 Gy was significant on multivariate analysis (*P* = 0.005).

**Conclusions:**

OS and radiation pneumonitis after SABR for stage I NSCLC can be predicted by staging PET SUV_max_ and ipsilateral mean lung dose, respectively.

## Background

Lung cancer is the leading cause of cancer death throughout the world and accounts for 28% of all cancer deaths in the United States [1]. Approximately 15%–20% of patients with non-small cell lung cancer (NSCLC) present with early or localized disease that could be treated surgically [2,3]. Stereotactic ablative radiotherapy (SABR), also known as stereotactic body radiotherapy (SBRT), can achieve local control rates exceeding 90% as well as promising survival rates in such cases when a biologically effective dose (BED) of more than 100 Gy is delivered to the planning target volume (PTV) [3-11]. SABR has emerged as a standard treatment option for stage I disease in patients who cannot undergo surgery for medical reasons [3-7] and for isolated recurrences of NSCLC [6,12,13]. However, the information about factors that may predict survival and pneunonitis after SABR is limited because of the heterogeneity of the patients and dose regimens [13-19].

In this report, we reported clinical outcome and used long-term follow-up data to identify potentially predictive factors for survival and pneumonitis among 130 patients with stage I NSCLC treated with SABR to 50 Gy delivered in 4 fractions over 4 consecutive days (BED 112.5 Gy).

## Methods

### Study design

We retrospectively analyzed 130 patients who had been prospectively enrolled in either a phase II clinical protocol on image-guided SABR (n = 46) or in our SABR program (n = 84) according to the same protocol guidelines at The University of Texas MD Anderson Cancer Center between February 2005 and December 2009. Reasons for not being enrolled in the phase II protocol included patient or insurance refusal, not having had the required brain magnetic resonance imaging (MRI) or computed tomography (CT), or not having signed the protocol-specific informed consent forms within the required time. All patients provided written informed consent to participate. Eligibility criteria included cytologically or biopsy-proven stage I NSCLC (T <5 cm, N0, M0) and inability or lack of desire to undergo surgery. Criteria for medical inoperability were having a baseline forced expiratory volume in 1 second (FEV1) or lung diffusion capacity <40% of predicted values or severe diabetes mellitus, cardiovascular disease, cerebral disease, or pulmonary hypertension. Thirty-four patients whose disease was considered borderline operable by thoracic surgeons had declined surgery. Disease in all patients was staged with chest CT and positron emission tomography (PET)/CT (Discovery ST; GE Healthcare, Milwaukee, WI) within 3 months before SABR and follow ups. The PET/CT scan condition was described previously (26). Lesions within 2 cm of the bronchial tree or mediastinal structures were considered central; all others were considered peripheral.

### Treatment planning

Techniques for patient immobilization and treatment planning are described elsewhere [6,12]. Briefly, patients were immobilized while supine with a customized vacuum immobilization bag extending from the head to the pelvis. Four-dimensional (4D) CT images were obtained in all cases. Gross tumor volumes (GTVs) were delineated by using maximum intensity projection of 4D CT and modified by visual verification at different breathing phases. The path of movement of the GTV during the respiratory cycle was the internal gross tumor volume (iGTV) [20]. The clinical target volume (CTV) was created by expanding the iGTV by 8 mm isotropically, with borders edited clinically. A 3-mm margin was added to CTV to account for set-up errors, thereby creating the PTV. No additional margins were used between the PTV and the block edge. Three-dimensional conformal SABR plans were optimized using 6 to 12 coplanar or non-coplanar 6-MV photon beams. SABR was prescribed to a dose of 50 Gy to the PTV between the 75% and 90% isodose lines, which had been created via Pinnacle calculation algorithms with heterogeneity correction, and delivered in 4 fractions over 4 consecutive days. Typically, the lower prescription isodose line was chosen when the proximity of critical normal structures mandated a compromise to the PTV, and therefore a higher dose to the tumor center and sharper dose gradients were required. Normal tissue dose-volume constraints were based on BED calculations and our previous clinical findings of the toxicity of SABR [6,12,21] and are shown in Table 1. Violations to the constraints for the spinal cord, esophagus, and brachial plexus were not allowed; constraints on other normal tissues were judged on the basis of clinical target coverage. Typically, when the tumor was close to a critical structure, a compromise in PTV coverage was considered acceptable. In any situation, however, the iGTV plus a margin of 5 mm was required to receive at least 95% of the prescribed dose. Patients with lesions very close/ abutting to critical structures and whose normal tissue dose volume constraints can’t be achieved were treated with different dose regimens. Day-to-day variations in patient placement were minimized by volumetric imaging of the treatment couch with either a CT-on-rails or a cone-beam CT system.

**Table 1:** Critical organ dose-volume limits for stereotactic ablative radiotherapy to 50 Gy given in 4 fractions

**Organ, Limit, and Volume**	**Maximum dose limits**
Esophagus	
D_max_	35 Gy
≤ 1 cm^3^	30 Gy
≤ 5 cm^3^	20 Gy
Brachial plexus	
D_max_	40 Gy
≤ 1 cm^3^	35 Gy
≤ 5 cm^3^	30 Gy
Trachea	
D_max_	45 Gy
≤ 1 cm^3^	35 Gy
≤ 5 cm^3^	30 Gy
Main bronchus and bronchial tree	
≤ 1 cm^3^	40 Gy
≤ 5 cm^3^	35 Gy
Heart	
≤ 1 cm^3^	40 Gy
≤ 5 cm^3^	35 Gy
Total lung volume*	
V_20 Gy(RBE)_	< 20% of total lung volume
V_10__Gy(RBE)_	< 30% of total lung volume
V_5__Gy(RBE)_	< 40% of total lung volume
Major vessels	
D_max_	45 Gy
≤ 1 cm^3^	40 Gy
≤ 5 cm^3^	35 Gy
Skin	
≤ 1 cm^3^	35 Gy
≤ 5 cm^3^	30 Gy
Chest wall	
≤ 10 cm^3^	45 Gy
≤ 30 cm^3^	35 Gy
Spinal cord	
D_max_	25 Gy
≤ 5 cm^3^	20 Gy

*Defined as right plus left lungs minus the gross tumor volume.

### Follow-up

Follow-up care consisted of CT imaging and clinical examination every 3 months for the first 2 years after SABR, every 6 months for the third year, and annually thereafter. All patients underwent posttreatment fluorodeoxyglucose (FDG) PET scans at MD Anderson for disease staging and at the first or second follow-up visit (median interval 4.3 months, range 2–7.6 months; the wide range reflected unexpectedly interrupted follow-up) and as clinically indicated thereafter. Rates and times of overall survival (OS), progression-free survival (PFS), local failure-free survival (LFFS), distant metastasis-free survival (DMFS), local failure, regional failure, and distant metastasis were calculated from the date of completion of SABR to the last available follow-up. The time of recurrence was the time at which the first image (PET/CT or CT) showed abnormalities. Local failure was defined as progressive abnormalities on CT images corresponding to one or more FDG-avid lesions on PET scans; positive biopsy findings within the PTV plus a 1-cm margin; or lesions that appeared in the same lobe after SABR. Recurrence appearing in different lobes was scored as distant metastasis. Regional failure was defined as intrathoracic lymph node relapse outside the PTV. Toxicities, including RP, were scored according to the National Cancer Institute Common Terminology Criteria for Adverse Events v3.0.

### Statistical analyses

Data were analyzed with SAS (SAS Institute, Cary, NC) statistical software, version 9.2. To analyze predictive factors for OS, PFS, LFFS, and DMFS after SABR, continuous variables such as age, FEV1, maximum standardized uptake value (SUV_max_) on staging PET scans, and GTV were discretely divided at the sample median and then analyzed as nominal categorical variables. We used the Kaplan-Meier method to estimate survival curves and the log-rank test to compare the curves. *P* values < 0.05 were considered statistically significant. Characteristics found to be significant by univariate analysis were then entered in multivariable Cox proportional hazards regression analysis.

To analyze predictive factors for RP, continuous variables such as age, FEV1, GTV, PTV, and dosimetric data were divided at the medians and analyzed as nominal categorical variables. Total lung volume was defined as right plus left lungs minus the GTV, and ipsilateral lung was defined as the lung containing the lesion to be treated minus the GTV. Comparisons were made with two-sided Pearson’s chi-square tests. *P* values <0.05 were considered statistically significant. Characteristics found to be significant by univariate analysis were then entered in a stepwise multiple binary logistic regression analysis to identify independent predictive factors.

## Results

### Patient characteristics, survival, and patterns of failure after SABR

**Figure 1:**
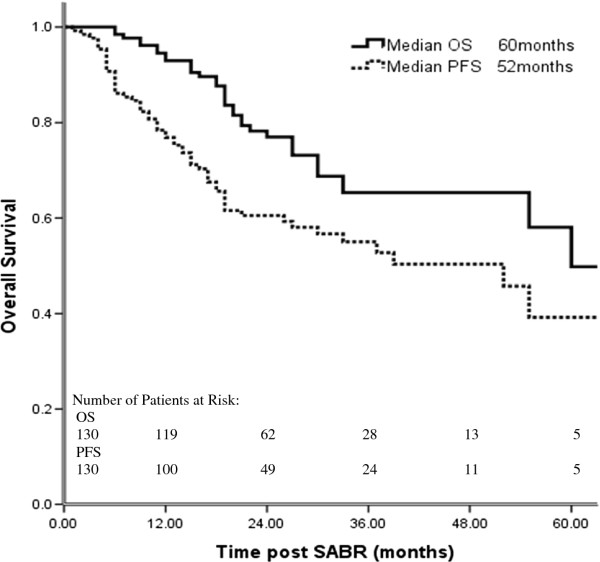
Overall survival (OS) and progression-free survival (PFS) for 130 patients treated with stereotactic ablative radiotherapy (SABR) for stage I NSCLC.

Characteristics of the 130 patients treated with SABR are listed in Table 2. At a median follow-up time of 26 months (range, 6–78 months), the median OS time for all patients was 60 months (55 months for patients with medically inoperable disease vs. >60 months [not reached] for those with borderline operable disease). One patient developed local failure concurrent with distant metastasis, and one patient developed isolated local failure that was salvaged surgically. At 2 years, the local control rate was 98.5%; the regional lymph node recurrence rate was 8.5% (11/130), and the isolated regional lymph node recurrence rate was 6.9% (9/130). Thirty patients (23.1%) developed DM, making it the dominant pattern of treatment failure. Overall and progression-free survival rates for all patients are illustrated in Figure 1. OS rates were 93.0% at 1 year, 78.2% at 2 years, and 65.3% at 3 years; the corresponding PFS rates were 78.4%, 60.5%, and 55.0%. With regard to disease control, LFFS rates were 93.7% at 1 year, 88.8% at 2 years, and 88.8% at 3 years; the corresponding DMFS rates were 89.1%, 79.4%, and 73.1%.

**Table 2:** Patient characteristics (n = 130)

**Characteristic**	**Value or No. of patients (%)**
Age, years	
Median	74
Range	48–91
FEV1, % of predicted	
Median	42
Range	15–123
Staging PET SUV_max_	
Median	6.20
Range	0.5–32.6
Gross tumor volume, cm^3^	
Median	9.6
Range	0.7–51.47
Planning target volume, cm^3^	
Median	73.2
Range	23.36–109.64
Sex	
Men	67 (51.5)
Women	63 (48.5)
COPD stage	
0-II	73 (56)
III-IV	57 (44)
History of other types of cancer	
Yes	37 (28.5)
No	93 (71.5)
ECOG performance status	
0 or 1	81 (62)
2 or 3	49 (38)
Lung cancer stage	
IA (T1)	112 (86)
IB (T2)	18 (14)
Lung cancer histology	
Squamous cell carcinoma	36 (28)
Adenocarcinoma	58 (45)
NSCLC not specified	36 (28)
Disease status	
Medically inoperable	96 (74)
Operable	34 (26)
Tumor location	
Peripheral	119 (91.5)
Central	11 (8.5)

Abbreviations: FEV1, forced expiratory volume in 1 second; PET, positron emission toography; SUVmax, maximum standardized uptake value; COPD, chronic obstructive pulmonary disease; ECOG, Eastern Cooperative Oncology Group; NSCLC, non-small cell lung cancer;

### Toxicity associated with SABR

No patient experienced grade 4 or 5 toxicity, even those with centrally located lesions. Chest wall pain was experienced by 12 patients (11 [8.5%] grade 2 and 1 [0.8%] grade 3), with median time to onset 8 months after SABR (range 0–27 months). Eight patients (6.2%) had grade 2 or 3 dermatitis (median onset time 2 months, range 0–10 months). Fifteen patients developed RP (12 [9.2%] grade 2 and 3 [2.3%] grade 3), with median time to onset 4 months (range 1–11 months). Only 2 patients (1.5%) developed esophagitis (both grade 1) with the esophageal dose-volume constraints used here. No definitive radiation-induced cardiovascular toxicity was noted.

### Predictors of OS and PFS after SABR

**Figure 2:**
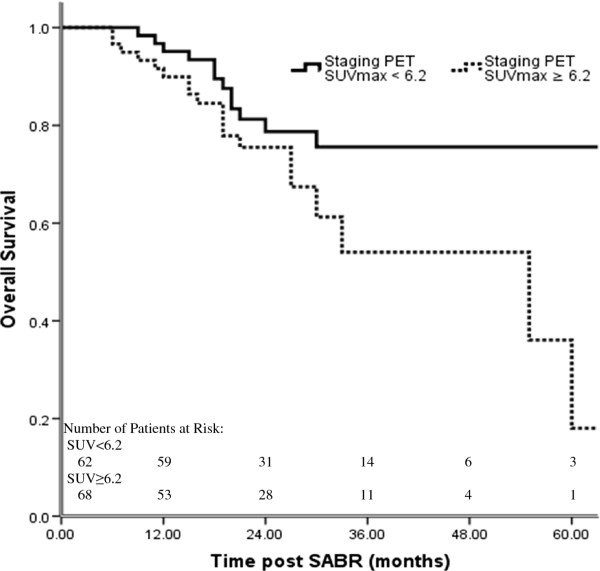
**Overall survival according to maximum standardized uptake value (SUV**_**max**_**) on staging PET/CT scans.**

Next we explored whether SUV_max_ or other variables could predict clinical outcomes after SABR for stage I disease. Univariate analysis revealed that staging PET SUV_max_ (dichotomized at the median 6.2, *P* = 0.028), Eastern Cooperative Oncology Group (ECOG) performance status (*P* = 0.037), tumor histology (*P* = 0.043), and whether the disease was considered medically operable or not (*P* = 0.036) were significantly associated with OS (Table 3); performance status and staging PET SUV_max_ were also associated with PFS after SABR. Multivariate Cox regression analysis showed that staging PET SUV_max_ was the only independent significant predictor of OS (hazard ratio [HR] 2.15; 95% confidence interval [CI] 1.06–4.34; *P* = 0.034): patients whose PET SUV_max_ at staging was less than the median 6.2 had higher rates of long-term OS than did those with staging PET SUV_max_ ≥6.2 (Figure 2, *P* = 0.034). No significant predictors were identified for LFFS and DMFS.

**Table 3:** Univariate analysis of predictive factors for overall and progression-free survival

	**Overall Survival**		**Progression-Free Survival**	
**Characteristic**	**HR (95% CI)**	***P*value**	**HR (95% CI)**	***P*Value**
Age, years				
≥ 74	1			
< 74	1.87 (0.93–3.78)	0.080	1.42 (0.83–2.44)	0.196
Sex				
Male	1			
Female	0.93 (0.48–1.82)	0.875	0.86 (0.51–1.47)	0.588
COPD stage				
0–II	1			
III–IV	0.75 (0.37-1.54)	0.413	0.66 (0.37-1.17)	0.594
History of other type of cancer				
Yes	1			
No	1.55 (0.78–3.09)	0.211	1.29 (0.74–2.24)	0.374
ECOG performance status				
0–1	1			
2–3	2.03 (1.04–3.95)	0.037	1.68 (0.99–2.85)	0.050
Lung cancer stage				
IA (T1)	1			
IB (T2)	1.28 (0.53–3.08)	0.586	1.11 (0.54–2.27)	0.776
Lung cancer histology				
Non-squamous	1			
Squamous	1.16 (1.01–1.34)	0.043	1.09 (0.97–1.22)	0.173
Staging PET SUV_max_				
< 6.2	1			
≥ 6.2	2.10 (1.01-4.33)	0.028	1.81 (1.05–3.10)	0.032
Gross tumor volume, cm^3^				
< 9.6	1			
≥ 9.6	1.55 (0.79–3.05)	0.204	1.42 (0.84–2.42)	0.195
Disease status				
Medically inoperable	1			
Operable	0.36 (0.14–0.94)	0.036	0.51 (0.26–1.02)	0.055
Tumor location				
Peripheral	1			
Central	0.97 (0.34–2.76)	0.954	0.89 (0.35–2.25)	0.809
Radiation pneumonitis				
No	1			
Yes	1.19 (0.65–2.16)	0.571	0.92 (0.43–1.94)	0.819

Abbreviations: HR, hazard ratio; CI, confidence interval; COPD, chronic obstructive pulmonary disease; ECOG, Eastern Cooperative Oncology Group; PET, positron emission tomography; SUVmax, maximum standardized uptake value.

### Risk factors for grade 2–3 RP

Univariate analysis of patient characteristics and dosimetric factors dichotomized at the medians (Table 4) revealed that many dosimetric variables (lung volumes receiving anywhere from 5 Gy to 40 Gy and mean lung doses [MLDs]) were associated with the incidence of grade 2–3 RP (*P* < 0.05) but that other patient characteristics (e.g., FEV1, tumor location, performance status, and GTV) were not. Multivariate binary logistic regression analysis with stepwise selection of variables found to be significant in univariate analysis showed that only an MLD to the ipsilateral lung ≥9.14 Gy (the median value) was associated with grade 2–3 RP (odds ratio [OR] 18.86; 95% CI 2.398–148.27; *P* = 0.005). Interestingly, when we used Cox regression analysis to take onset time of pneumonitis into consideration, ipsilateral lung V40 become the most significant predictor for grade 2–3 RP, indicating high dose (40 Gy) may be correlated with grade 2–3 RP developed within certain time after SABR.

**Table 4:** Univariate analysis of patient and dosimetric characteristics and risk of radiation pneumonitis (n = 130)


**Characteristic**	**Grade 0–1 RP**	**Grade 2–3 RP**	***P*Value**
	**No. of Patients (%)**	**No. of Patients (%)**	
Sex			0.882
Male	59 (51.3)	8 (53.3)	
Female	56 (48.7)	7 (46.7)	
Age			0.310
≥74 years	62 (53.9)	6 (40.0)	
<74 years	53 (46.1)	9 (60.0)	
COPD stage			0.154
0–II	62 (53.9)	11 (73.3)	
III–IV	53 (46.1)	4 (26.7)	
History of other type of cancer			0.440
Yes	34 (29.6)	3 (20.0)	
No	81 (70.4)	12 (80.0)	
ECOG score before SABR			0.711
0–1	71 (61.7)	10 (66.7)	
2–3	44 (38.3)	5 (33.3)	
Gross tumor volume, cm^3^			0.375
≥9.6	55 (47.8)	9 (60.0)	
<9.6	60 (52.2)	6 (40.0)	
Planning target volume, cm^3^			0.151
≥73.2	54 (47.0)	10 (66.7)	
<73.2	61 (53.0)	5 (33.3)	
Tumor location			0.471
Peripheral	106 (92.2)	13 (86.7)	
Central	9 (7.8)	2 (13.3)	
Total lung volume*			
V_5_			< 0.001
≥ 20.2%	51 (44.3)	14 (93.3)	
< 20.2%	64 (55.7)	1 (6.7)	
V_10_			< 0.001
≥ 14.3%	51 (44.3)	14 (93.3)	
< 14.3%	64 (55.7)	1 (6.7)	
V_15_			0.003
≥ 11.0%	52 (45.2)	13 (86.7)	
< 11.0%	63 (54.8)	2 (13.3)	
V_20_		0.055	
≥ 8.5%	54 (47.0)	11 (73.3)	
< 8.5%	61 (53.0)	4 (26.7)	
V_25_			0.055
≥ 6.4%	54 (47.0)	11 (73.3)	
< 6.4%	61 (53.0)	4 (26.7)	
V_30_			0.003
≥ 5.0%	52 (45.2)	13 (86.7)	
< 5.0%	63 (54.8)	2 (13.3)	
V_35_			0.003
≥ 3.9%	52 (45.2)	13 (86.7)	
< 3.9%	63 (54.8)	2 (13.3)	
V_40_			0.013
≥ 3.1%	53 (46.1)	12 (80.0)	
< 3.1%	62 (53.9)	3 (20.0)	
Mean dose to total lung volume			0.013
≥ 5.05 Gy	53 (46.1)	12 (80.0)	
< 5.05 Gy	62 (53.9)	3 (20.0)	
Ipsilateral lung volume†			
V_5_			< 0.001
≥ 37.7%	51 (44.3)	14 (93.3)	
< 37.7%	64 (55.7)	1 (6.7)	
V_10_			< 0.001
≥ 28.5%	51 (44.3)	14 (93.3)	
< 28.5%	64 (55.7)	1 (6.7)	
V_15_			< 0.001
≥ 21.9%	51 (44.3)	14 (93.3)	
< 21.9%	64 (55.7)	1 (6.7)	
V_20_			< 0.001
≥ 16.9%	51 (44.3)	14 (93.3)	
< 16.9%	64 (55.7)	1 (6.7)	
V_25_			0.003
≥ 13.1%	52 (45.2)	13 (86.7)	
< 13.1%	63 (54.8)	2 (13.3)	
V_30_			0.013
≥ 10.4%	53 (46.1)	12 (80.0)	
< 10.4%	62 (53.9)	3 (20.0)	
V_35_			0.003
≥ 8.1%	52 (45.2)	13 (86.7)	
< 8.1%	63 (54.8)	2 (13.3)	
V_40_			0.013
≥ 6.3%	53 (46.1)	12 (80.0)	
< 6.3%	62 (53.9)	3 (20.0)	
Mean dose to ipsilateral lung volume			< 0.001
≥9.14 Gy	51 (44.3)	14 (93.3)	
<9.14 Gy	64 (55.7)	1 (6.7)	

* Defined as right plus left lungs minus the gross tumor volume.

† Defined as the lung containing the lesion to be treated, minus the gross tumor volume.

Abbreviations: RP, radiation pneumonitis; COPD, chronic obstructive pulmonary disease; ECOG, Eastern Cooperative Oncology Group; SABR, stereotactic ablative radiotherapy; Vx, volume of lung receiving =x Gy; GTV, gross tumor volume; MLD, mean lung dose.

## Discussion

We found that SABR to a dose of 50 Gy delivered in 4 fractions (BED 112.5 Gy) produced a 2-year local control rate of 98.5%, a median OS time of 60 months, and minimal toxicity (minimal grade 3 and no grade 4 or 5). SUV_max_ on the staging PET/CT scan was the only predictor of OS, with SUV_max_ less than the median 6.2 being associated with better survival. The MLD to the ipsilateral lung (i.e., the lung containing the lesion to be treated, minus the GTV) was the only significant predictor of grade 2 or 3 RP. Among 130 patients, only two (<2%) experienced LF, one of which occurred simultaneously with DM. The thoracic lymph node recurrence rate of 8.5% was consistent with most reported findings [3-11], and DM remained the dominant pattern of failure. This finding, common in other studies as well [3-7,9], underscores the need for novel systemic treatments to reduce the incidence of distant failure. Molecular markers may also be helpful for identifying patients who may benefit from adjuvant chemotherapy.

Having other predictive tools in addition to traditional factors such as age, disease stage, performance status, tumor histology, and comorbidities to predict outcome before therapy is begun would be valuable both for the choice of initial treatment and for identifying which patients might benefit from additional systemic therapy. Several surgical series [22-25] showed that pretreatment SUV_max_ had predictive value in stage I NSCLC treated surgically; one of these studies, an analysis of 136 patients, found that a pretreatment SUV_max_ >5.5 predicted worse recurrence and survival [23]. However, information on SUV and SABR remains very limited at this time [14-16,26]. Hoopes et al. [14] retrospectively evaluated the predictive value of PET SUV_max_ in a prospective phase I/II dose escalation clinical trial in which SABR was given to 58 patients at doses of 24 to 72 Gy in 3 fractions. Local control rates in that trial ranged from <70% to >95% for the various dose groups, and pretreatment PET SUV_max_ was not found to predict local control or survival. Another retrospective study by Burdick and colleagues [16] showed that pretreatment SUV_max_ did not predict regional failure, distant failure, or survival; however, the 72 patients in that study had also been treated with a wide range of radiation doses (60 Gy in 3 fractions, 50 Gy in 5 fractions, or 50 Gy in 10 fractions), and only 68.1% of patients had had biopsy-proven NSCLC.

The relative strengths of our study were our relatively large population (n = 130) and our inclusion of only patients with biopsy-proven, PET/CT-determined stage I (T1N0M0, T <5 cm) NSCLC who had all been treated with the same dose and who all underwent PET/CT both before and after treatment at the same institution. Our multivariate analyses indicated that having a staging PET SUV_max_ level >6.2 predicted worse OS, and patients with this feature may benefit from systemic therapy to reduce the likelihood of distant failure, which still remains problematic. The predictive value of PET SUV may well depend on the dose regimen used and perhaps some patient characteristics that we did not consider. Additional studies are needed to validate our observations.

As we and others reported before, PET SUVs measured after SABR may be useful for detecting recurrence (19, 26). In the current study, the staging PET SUV_max_ levels for the 2 patients who developed local recurrence were 1.8 and 6.5 but had increased to 9.8 and 7.2, respectively, by 1 year after SABR. However, among the other 128 patients who did not experience local recurrence in this study, 32 patients had a SUV_max_ >3 and 8 patients had a SUV_max_ >5 within 6 months after SABR. Thus it seems likely that PET images obtained within 6 months after SABR may have a high false-positive rate. Indeed, we and others have noted that PET images with SUV >5 more than 6–12 months after SABR could indicate possible local recurrence, but biopsy is still recommended for confirmation [−25, 26], particularly when salvage surgery is planned [27].

The most common side effect of SABR in our study was chest-wall pain (12 patients, or 9.3%). A previous study from our group showed that limiting the chest-wall V_30_ to < 30 cm^3^ reduced the incidence of chest-wall pain to 5% [21]. However, for lesions next to the chest wall, we recommend that >95% of the GTV plus a 5-mm margin receive at least the full prescribed dose, even if the chest-wall dose exceeds 35 Gy to 30 cm^3^. In our practice, 35 Gy to 50 cm^3^ is allowed for lesions close to the chest wall.

RP can be a severe or even fatal side effect of irradiation for lesions within 2 cm of the bronchial tree treated with 54 Gy delivered in 3 fractions [4]. Reports of dose-volume analyses in SABR-induced RP have been limited [13,17,18,28-30]. Barriger and others reported correlations between total lung MLD (<4 Gy vs. >4 Gy), lung V_20_ (<4% vs. >4%) and grade 2–4 RP among patients treated with SABR to total doses of 42–60 Gy given in 8- to 20-Gy fractions [31]. Matsuo found the association between V25 and symptomatic RP after SABR (17) . With our dose regimen (50 Gy in 4 fractions), our normal-tissue dose-volume constraints (Table 1), and our use of 4D CT-based treatment planning and volumetric on-board image-guided SABR delivery, we did not observe any grade 4–5 RP. We saw no difference in RP between central versus peripheral lesions when normal tissue dose volume constraints were respected and inappropriate cases were excluded, and only 3 patients (2.3%) experienced grade 3 RP. Interestingly, only MLD to the ipsilateral lung was significantly associated with RP in multivariate analysis; among the 65 patients with an ipsilateral MLD ≥9.14 Gy, 14 had grade 2–3 RP (21.5%), whereas among the 65 with an ipsilateral MLD <9.14 Gy, only 1 (1.5%) had grade 2–3 RP (*P* < 0.001). This finding is consistent with those of Guckenberger and colleagues, who also reported a correlation between irradiated ipsilateral lung volume and SABR-induced RP [30]. In addition, ipsilateral V40 appears to be correlated with grade 2–3 RP when the onset times of RP were considered. The specific dose cutoffs may be different using different dose regimens. Our cutoffs should be considered only when the same or similar SABR dose regimens are used. To minimize the MLD to the ipsilateral lung, one should consider using optimal image guidance to reduce the set-up margin; prescribing the dose to the lower isodose line rather than the higher one; and not using an additional margin between the PTV to the block edge.

## Abbreviations

4D: Four-dimensional; BED:Biologically equivalent dose; CI: Confidence interval; CT: Computed tomography; DMFS: Distant metastasis-free survival; ECOG: Eastern cooperative oncology group; FDG: Fluorodeoxyglucose; FEV1: Forced expiratory volume in 1 second; GTV: Gross tumor volume; HR: Hazard ratio; iGTV: Internal gross tumor volume; LFFS: Local facilure-free survival; MLD: Mean lung dose; MRI: Magnetic resonance imaging; NSCLC: Non-small cell lung cancer; OR: Odds ratio; OS: Overall survival; PET: Positron emission tomography; PFS: Progression-free survival; PTV: Planning target volume; RP: Radiation pneumonitis; SABR: Stereotactic ablative radiotherapy; SUV_max_: Maximum standardized uptake value.

## Competing interests

The authors declare that they have no competing interests.

## Authors’ contributions

JYC conceived the study, oversaw the study design and data collection, and wrote the manuscript; HL helped to conceive and coordinate the study, to collect data, and to write the manuscript; JYC and PB designed the treatment plans and supervised their delivery; JYC and HL carried out the data and statistical analysis; and RK, ZL, JW, RJM, JAR, and SGS provided data and participated in the coordination and design of the study. All authors read and approved the final manuscript.

## Funding

Dr. Chang received a Research Scholar Award from the Radiological Society of North America and a Career Development Award from MD Anderson Cancer Center’s Specialized Programs of Research Excellence in Lung Cancer from the National Cancer Institute (P50 CA70907). This research was also supported by Cancer Center Support Grant CA016672 to MD Anderson.
